# Androgen receptor in Sertoli cells regulates DNA double-strand break repair and chromosomal synapsis of spermatocytes partially through intercellular EGF-EGFR signaling

**DOI:** 10.18632/oncotarget.7916

**Published:** 2016-03-04

**Authors:** Su-Ren Chen, Xiao-Xia Hao, Yan Zhang, Shou-Long Deng, Zhi-Peng Wang, Yu-Qian Wang, Xiu-Xia Wang, Yi-Xun Liu

**Affiliations:** ^1^ State Key Laboratory of Stem Cell and Reproductive Biology, Institute of Zoology, Chinese Academy of Sciences, Beijing, People's Republic of China; ^2^ University of Chinese Academy of Sciences, Beijing, People's Republic of China

**Keywords:** androgen receptor, Sertoli cells, meiosis, synapsis, DNA double-strand breaks

## Abstract

Spermatogenesis does not progress beyond the pachytene stages of meiosis in Sertoli cell-specific AR knockout (SCARKO) mice. However, further evidence of meiotic arrest and underlying paracrine signals in SCARKO testes is still lacking. We utilized co-immunostaining of meiotic surface spreads to examine the key events during meiotic prophase I. SCARKO spermatocytes exhibited a failure in chromosomal synapsis observed by SCP1/SCP3 double-staining and CREST foci quantification. In addition, DNA double-strand breaks (DSBs) were formed but were not repaired in the mutant spermatocytes, as revealed by γ-H2AX staining and DNA-dependent protein kinase (DNA-PK) activity examination. The later stages of DSB repair, such as the accumulation of the RAD51 strand exchange protein and the localization of mismatch repair protein MLH1, were correspondingly altered in SCARKO spermatocytes. Notably, the expression of factors that guide RAD51 loading onto sites of DSBs, including TEX15, BRCA1/2 and PALB2, was severely impaired when either AR was down-regulated or EGF was up-regulated. We observed that some ligands in the epidermal growth factor (EGF) family were over-expressed in SCARKO Sertoli cells and that some receptors in the EGF receptor (EGFR) family were ectopically activated in the mutant spermatocytes. When EGF-EGFR signaling was repressed to approximately normal by the specific inhibitor AG1478 in the cultured SCARKO testis tissues, the arrested meiosis was partially rescued, and functional haploid cells were generated. Based on these data, we propose that AR in Sertoli cells regulates DSB repair and chromosomal synapsis of spermatocytes partially through proper intercellular EGF-EGFR signaling.

## INTRODUCTION

The production of haploid gametes by meiosis is a cornerstone of sexual reproduction and the maintenance of genome integrity. Meiosis errors and genetic disruptions can cause aneuploidy and infertility [1, 2]. Prophase I of meiosis I is unique in that it is elongated, and this stage can be divided into the leptotene, zygotene, pachytene, diplotene, and diakinesis substages. During these substages of prophase I, the chromosomes undergo numerous changes that enable homologous recombination and the exchange of genetic information between non-sister chromatids. Once paired, homologous chromosomes are connected by the synaptonemal complex (SC), which consists of the central element, axial/lateral elements, and transverse filaments [3]. The initial steps of homologous recombination involve introduction of double-stranded breaks (DSBs) into the genome by the type II-like topoisomerase SPO11 [4]. The activation of ATM and ATR by SPO11-induced DSBs triggers the phosphorylation of a large set of substrates, including the checkpoint mediator kinases CHK1 and CHK2 as well as histone H2AX, to activate the DNA damage response [5]. DSBs are processed to produce single-stranded DNA (ssDNA) ends that can be used to probe for homology through strand invasion with the RAD51 and DMC1 recombinases [6]. TEX15, BRCA1, BRCA2 and PALB2 have been suggested to mediate RAD51 and DMC1 loading onto sites of DSBs [7–10]. After the homology search, the SC forms and connects the axes of aligned homologues to induce crossover (CO) formation [11]. At the pachytene stage of meiosis, DSBs are repaired using homologous sequence, and the DSB site becomes undetectable on autosomes and restricted to the XY body [12]. Failure of key events during meiotic prophase I contributes to the primary cause of meiotic arrest [13].

Genetic studies in mice demonstrated that meiosis is one of the most important steps that is controlled by androgens. In classical testosterone signaling, androgens exert their genomic effects via the androgen receptor (AR) [14]. Cytoplasmic AR, when bound by androgens, translocates to the nucleus and binds to androgen response elements (ARE) within androgen-responsive genes [15]. Very recently, Toocheck et al. suggest that testosterone acts through a non-classical pathway via the androgen receptor to rapidly activate kinases that are known to regulate spermatogenesis [16]. Testicular AR is expressed in Sertoli cells, Leydig cells, and peritubular myoid cells (PTMs) [17]. Various AR knockout (ARKO) mouse models have been developed to study the physiological and cellular roles of AR in spermatogenesis (reviewed in [18]). Male global ARKO mice, which share a similar phenotype with humans with androgen insensitivity syndrome (AIS) and with the testicular feminization (*Tfm*) mouse, exhibit a typical female external appearance and an early meiosis arrest phenotype [19, 20]. In the absence of AR in Sertoli cells, murine spermatogenesis does not progress beyond the pachytene or diplotene stages of meiosis [21, 22]. Normal spermatogenesis and fertility were observed in germ cell-specific AR knockout mice [23]. AR function in Leydig cells and PTMs is essential for the maintenance of appropriate Leydig cell steroidogenic function and PTM contractility, respectively, and the subsequent effects on Sertoli cells support the final differentiation of spermatozoa [23–26]. Currently, more than 1029 different AR missense mutations have been reported to cause AIS, contributing to approximately 2% of unselected male infertility [27]. Germ cells lack AR expression, but they depend on androgens for meiosis, indicating that androgens affect meiosis by acting on somatic cells. Within the seminiferous tubules, only Sertoli cells express the AR, and thus, these cells are considered the mediators of androgen action on meiocytes. However, the molecular mechanism by which Sertoli cells transduce the androgenic stimulus to spermatocytes has not been identified yet.

Among the various growth factors produced by Sertoli cells, the epithelial growth factor (EGF) family garnered our attention because they promote meiotic initiation in the neonatal mouse testis [28] and transcriptionally or post-transcriptionally respond to androgens [29, 30]. There are multiple related ligands in the EGF family, including EGF, heparin-binding EGF (HBEGF), transforming growth factor alpha (TGFα), amphiregulin (AREG), epiregulin (EREG), epigen (EPGN), betacellulin (BTC) and neuregulins 1–4 (NRG1-4) [31]. Receptors in the EGFR family, including EGFR (ERBB1) and ERBB2-4, function by ligand-dependent dimerization and activation of the tyrosine kinase in the cytoplasmic domain [32]. We hypothesis that Sertoli cells transduce the androgenic stimulus to spermatocytes partially via EGF-EGFR signaling for the following cues: (i) EGF family growth factors are secreted by Sertoli cells, and EGF receptors are present on the surface of spermatocytes [33, 34]; (ii) there is a similar meiosis arrest phenotype between SCARKO and EGF transgenic males [21, 22, 35]; (iii) two previous microarray data indicate that *AR*-null testes express elevated levels of several EGF-EGFR signaling molecules, including *Egf*, *Tgf*α, *Btc* and *Erbb4* (GEO2R analysis of GEO database: GSE2259 and GSE20918) [36, 37]; and (iv) EGFR regulates ATM activation, homologous recombination, and DNA repair in response to irradiation [38].

In the absence of AR expression in Sertoli cells, murine spermatogenesis does not progress beyond meiosis [21, 22]. Here, we extend these findings by determining the reasons for meiosis arrest in SCARKO spermatocytes using spermatocyte surface spreads. We found that SCARKO spermatocytes exhibited failed chromosomal synapsis and DSB repair. Importantly, we observed that EGF-EGFR signaling in testes was abnormally high in the absence of Sertoli cell AR. In addition, AR inhibition or EGF up-regulation could attenuate RAD51 and DMC1 expression as well as the protein levels of factors (TEX15, BRCA1/2 and PALB2) that guide RAD51 loading onto sites of DSBs. Finally, organ culture of SCARKO testes with the EGFR phosphorylation-inhibitor AG1478 (200 μM) partially restored meiosis and generated haploid sperm. Taken together, we conclude that EGF-EGFR signaling, at least in part, mediates Sertoli cell AR effects on meiocytes.

## RESULTS

### Aberrant chromosomal synapsis in SCARKO spermatocytes

**Figure 1:**
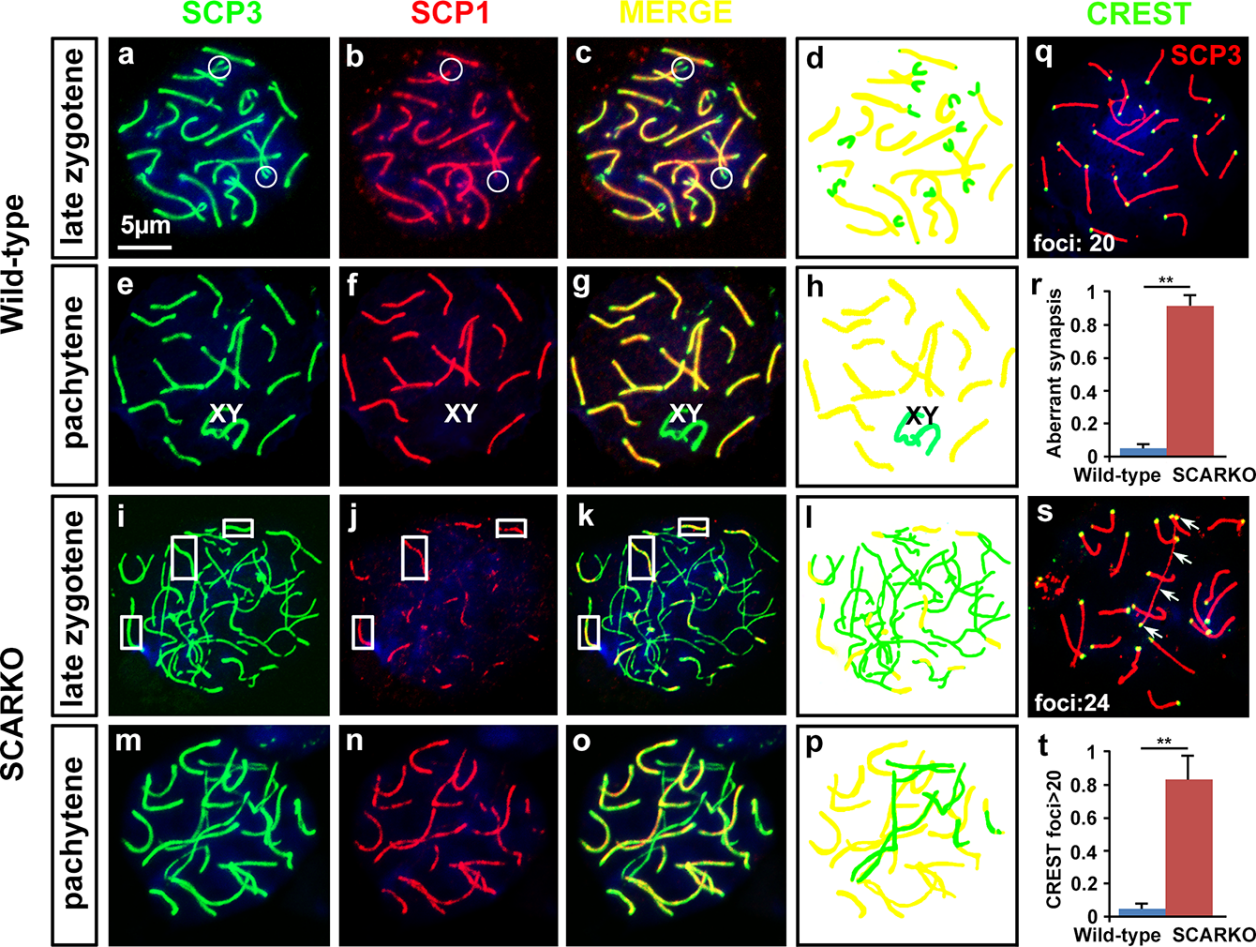
Defective synapsis of homologous chromosomes in SCARKO spermatocytes Representative chromosome spreads of spermatocytes at postnatal day 21 labeled with anti-SCP3 (green) and anti-SCP1 (red) antibodies. The late zygotene (a-c and i-k) and pachytene (e-g and m-o) stages of meiotic prophase I spermatocytes are shown. In the late zygotene stage, disconnected segments were only observed at the termini of pairing chromosomes (circles) in wild-type spermatocytes (a-c), while only some segments (rectangles) showed co-localization of SCP3 and SCP1 in SCARKO spermatocytes (i-k). Complete bivalents were detected at the pachytene stage in wild-type spermatocytes (e-g). However, incomplete pairing of homologs as well as univalent chromosomes were present in mutant spermatocytes (m-p). The number of meiocytes with defective synapsis was significantly different in SCARKO spermatocytes and control spermatocytes (**p < 0.01) (r). d, h, l and p show the differing morphologies of the chromosomes (yellow: paired chromosomes; green: unpaired chromosomes). Chromosome spreads of spermatocytes were immunostained for CREST autoimmune serum (green), which stains centromeres, and anti-SCP3 (red). Wild-type pachytene stage spermatocytes exhibited 20 CREST foci implying full synapsis of homologous chromosomes (q). Note the increase in CREST foci in mutant spermatocytes at the pachytene-like stage (s). Arrows in s indicate univalent chromosomes with two CREST foci. The percentage of spermatocytes containing univalent chromosomes (> 20 CREST foci) was counted (**p < 0.01) (t). Scale bar, 5 μm.

Previous studies demonstrated that SCARKO leads to spermatogenesis arrest specificly at the diplotene primary spermatocyte stage prior to accomplishing the first meiotic division [21, 22]. To determine the cause of this meiotic arrest and to gain mechanistic insight into this defect in SCARKO spermatocytes, we examined the assembly of the synaptonemal complex (SC) by surface spread analysis of spermatocytes. SC morphology in spermatocyte nuclei can be assessed by immunostaining of SC protein 1 (SCP1) and SCP3, which form the central and axial/lateral elements of the SC [3]. Using SCP1/SCP3 double-staining of wild-type pachytene spermatocytes, we observed perfect colocalization of SCP1 and SCP3 around the whole SC (Figure 1 g, h; yellow); in the corresponding SCARKO spermatocytes, synapsis occurred in some regions, but a significantly higher number of unsynapsed or partially synapsed chromosomes was observed (Figure 1 o, p; green, r). To confirm the presence of univalent chromosomes, we used CREST autoimmune serum, which stains centromeres, and anti-SCP3 to stain chromosomes at the pachytene stage (Figure 1 q, s). We quantified the number of CREST foci on homologues in SCARKO spermatocytes compared to wild-type spermatocytes. We found that approximately 85% of SCARKO diplotene spermatocytes (50 cells counted from 3 males) contained univalent chromosomes (> 20 CREST foci), while very few univalent chromosomes were observed in wild-type diplotene spermatocytes (48 cells counted from 3 males) (Figure 1 t). These data are consistent with the unsynapsed or partially synapsed chromosomes observed by SCP1/SCP3 double-staining (Figure 1 a–p, r). Collectively, these results indicate that Sertoli AR signal is required for spermatocytes to complete chromosomal synapsis.

### DSBs are formed, but not repaired, in SCARKO spermatocytes

**Figure 2:**
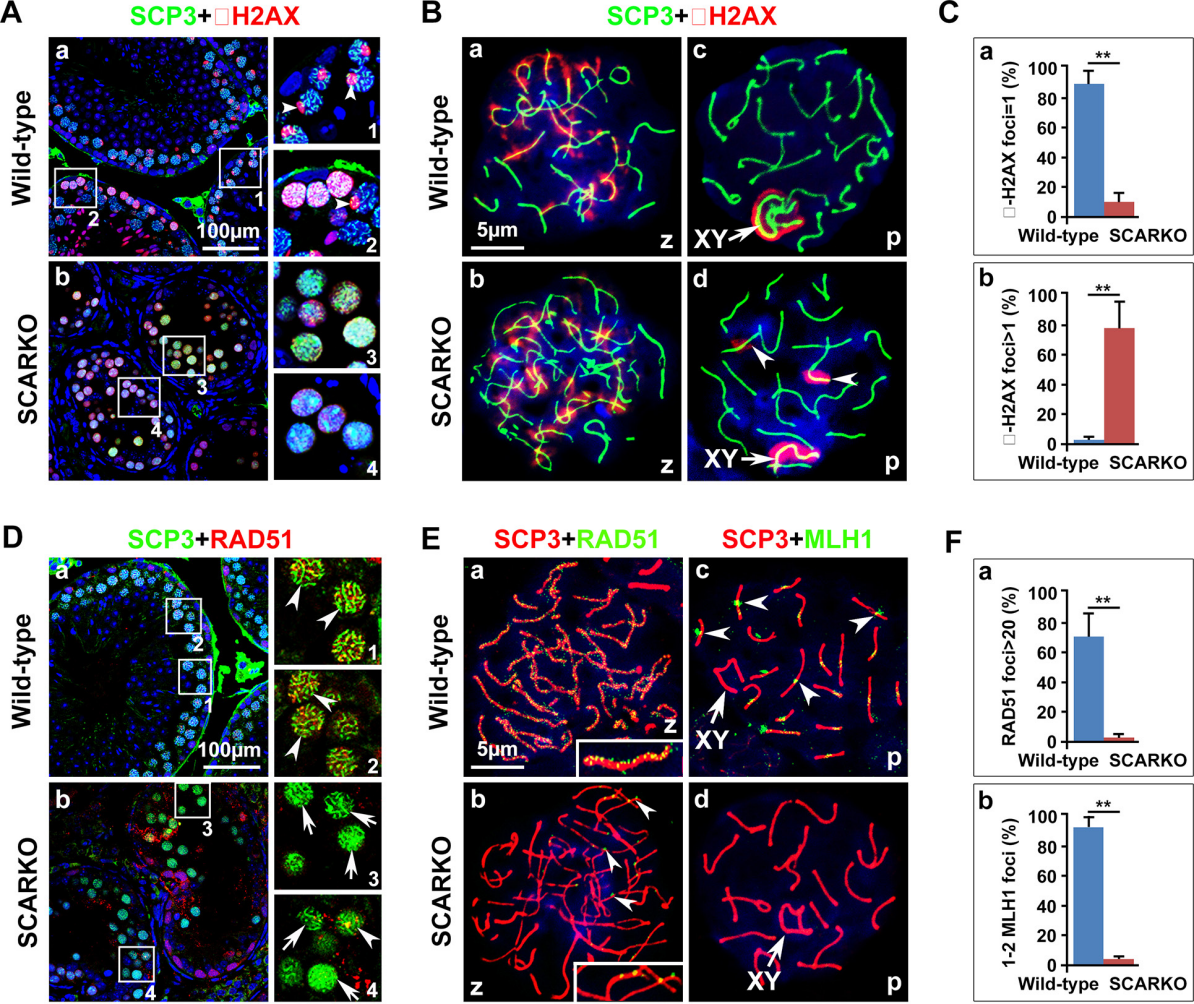
Meiotic DSBs are generated but not repaired in SCARKO spermatocytes (**A**) Testis sections from postnatal day 21 wild-type (a) and SCARKO (b) males were stained with anti-SCP3 (green) and anti-γ-H2AX (red) antibodies. γ-H2AX foci disappeared at the pachytene stage, except in the XY body in wild-type spermatocytes (pointed shape distribution, arrowheads; 1 and 2 are partial enlarged drawings of a), but no preferential localization to the sex chromosomes was observed in the SCARKO spermatocytes at this stage (diffuse distribution; 3 and 4 are partial enlarged drawings of b). Scale bar, 100 μm. (**B**) Chromosome spreads of wild-type and SCARKO spermatocytes at postnatal day 21 after immunostaining for SCP3 (green) and γ-H2AX (red). In response to DSBs, γ-H2AX foci appeared at the damage sites during the zygotene stage in both the wild-type (a) and the SCARKO testes (b). γ-H2AX staining localized preferentially to the XY sex chromosome (arrows) in the wild-type pachytene stage spermatocytes (c) but was sustained on asynapsed autosomal homologs (arrowheads) in mutant spermatocytes (d). Scale bar, 5 μm. (**C**) The percentage of γ-H2AX foci=1 (a) or > 1 (b) (**p < 0.01). (**D**) Testis sections from postnatal day 21 wild-type (a) and SCARKO (b) males were stained with anti-SCP3 (green) and anti-RAD51 (red) antibodies. 1, 2 and 3, 4 are partial enlarged drawings of a and b, respectively. Arrowheads indicate spermatocytes expressing abundant RAD51, and the arrows point to spermatocytes with insufficient RAD51 expression. Scale bar, 100 μm. (**E**) Chromosome spreads of wild-type (a, c) and SCARKO (b, d) spermatocytes at postnatal day 21 after immunostaining for anti-SCP3 (green) and anti-RAD51 (a, b; red) or MLH1 (c, d; red). In the zygotene stage, RAD51 foci were seldom observed in mutant spermatocytes (arrowheads in b), but was abundant in wild-type spermatocytes (see enlarged view in a). Arrowheads in c indicate MLH1 foci on each chromosome within wild-type spermatocytes. Scale bar, 5 μm. (**F**) A dramatic reduction (**p < 0.01) was observed in the number of RAD51 foci per zygotene spermatocyte (a) and MLH1 foci per pachytene spermatocyte (b) in SCARKO mice. The data in C and F represent the mean ± SEM from approximately 50 spermatocytes at the appointed time for each genotype. z, zygotene; p, pachytene.

To investigate the mechanism that mediates the synapsis defects in mutant spermatocytes, we examined whether DNA double-strand break (DSB) formation and meiotic recombination repair were impaired. During meiosis, γ-H2AX (a phosphorylated form of histone H2AX) is an important protein involved in the recognition of and signaling from DSBs. In response to DSBs, γ-H2AX foci responded equally well and appeared at the damage sites during the zygotene stage in both the wild-type and the SCARKO testes, with the same staining pattern and comparable intensity (Figure 2B a, b). Consistent with previous reports, at the pachytene stage of wild-type spermatocytes, γ-H2AX staining was undetectable on autosomes and restricted to the largely asynapsed XY body (Figure 2A a; Figure 2B c). Conversely, γ-H2AX staining of mutant spermatocytes was sustained in asynapsed autosomal homologs at the pachytene stage (Figure 2A b; Figure 2B d; arrowheads) at a significantly higher proportion than in wild-type spermatocytes (Figure 2C). All components of the DNA-dependent protein kinase (DNA-PK) complex are present in the nuclei of spermatocytes and DNA-PK functions in DNA repair [39, 40]. To further support our immunofluorescence staining results, we examined DNA-PK activity in extract of wild-type and SCARKO spermatocytes. Peptide kinase activity was observed in the extract of wild-type spermatocytes. In contrast, SCARKO spermatocytes contained no DNA-PK activity (Figure S1). Collectively, these data suggest that DSBs form normally, but cannot be properly repaired without AR paracrine signaling.

We next monitored the recombination process to ascertain why DSBs are not repaired in SCARKO spermatocytes. RAD51 binds to DSBs and plays critical roles in catalyzing homologous pairing, DNA strand exchange and DSB repair (reviewed in [41]). The number of RAD51 foci was markedly lower in mutant spermatocytes compared to wild-type controls (Figure 2D; Figure 2E a, b). We found that approximately 70% of zygotene wild-type spermatocytes contained more than 20 RAD51 foci. In contrast, the percentage of spermatocytes from SCARKO testes that exhibited > 20 RAD51 foci was extremely reduced (Figure 2F a). Moreover, we used an antibody to the mismatch repair protein, MLH1, to evaluate the later stages of DSB repair and the formation of meiotic crossovers in SCARKO spermatocytes. While each homologue in wild-type spermatocytes should have at least one crossover (MLH1 foci), it was not surprising that we hardly observed MLH1 foci in SCARKO spermatocytes (Figure 2E c, d; Figure 2F b). Altogether, these data indicate that the depletion of AR from Sertoli cells disrupts meiotic recombination repair in spermatocytes likely by abolishing the recruitment of RAD51 recombinase.

### Hyperactivation of EGF-EGFR signaling in SCARKO testis

**Figure 3:**
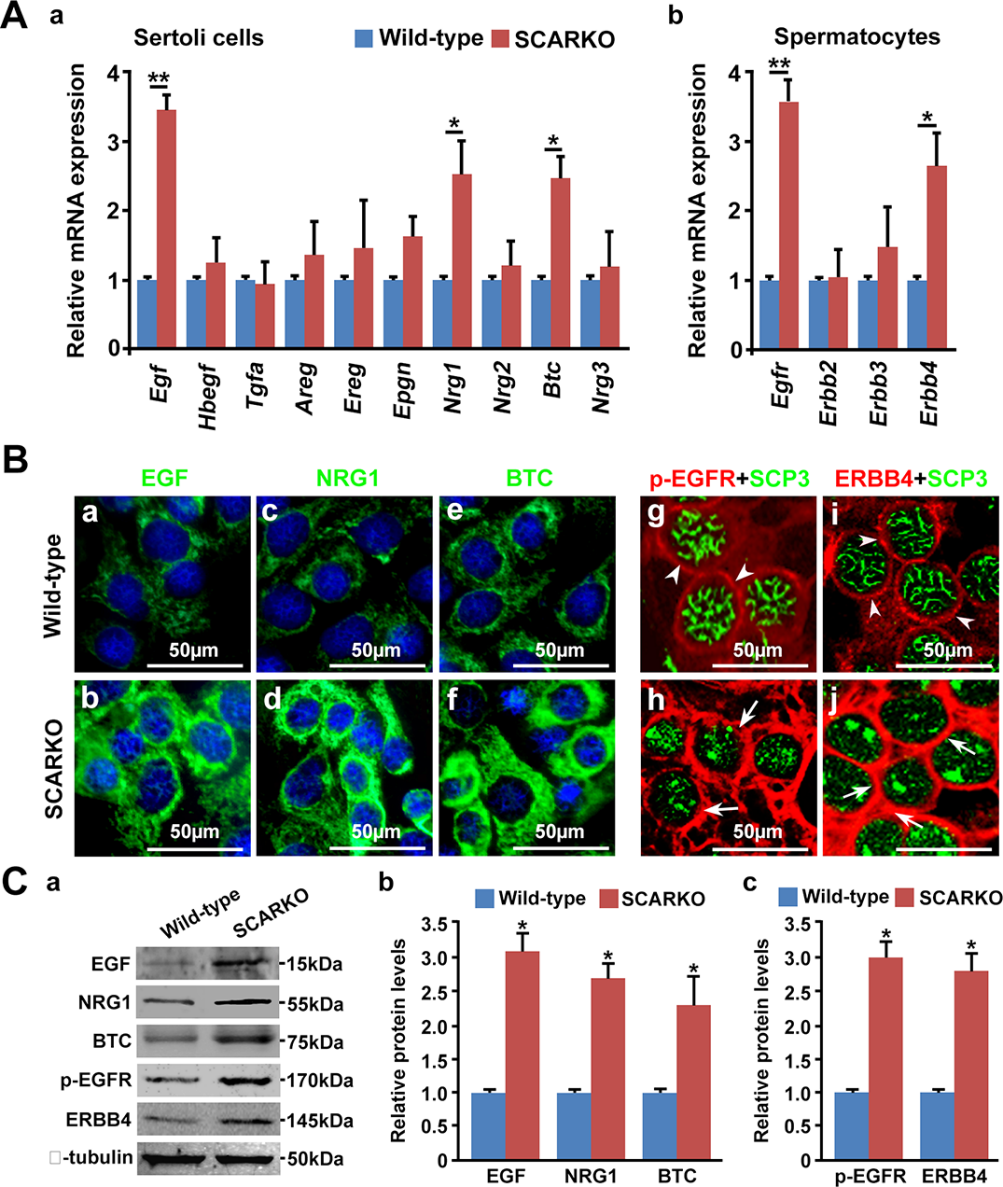
EGF-EGFR signaling is hyperactivated in the SCARKO testes (**A**) Relative expression of EGF family ligands in Sertoli cells and their receptors in spermatocytes. Sertoli cells were isolated from postnatal day 15 testes, and the mRNA levels of EGF family ligands, including *Egf*, *Hbegf*, *Tgfα*, *Areg*, *Ereg*, *Epgn*, *Btc* and *Nrg1-3*, were measured (a). Spermatocytes were purified from postnatal day 15 testes, and the relative expression of EGF family receptors, including *Egfr*, *Erbb2*, *Erbb3* and *Erbb4*, was measured (b). *Gapdh* served as the internal control gene. The data are expressed as the mean±SEM. *p < 0.05, **p < 0.01. (**B**) Representative cellular expression of EGF family ligands, such as EGF, NRG1 and BTC (a-f; green), in cultured Sertoli cells and their receptors (p-EGFR and ERBB4) (g-j; red) in SCP3 (green)-positive spermatocytes from wild-type (a, c, e, g, i) and SCARKO testes (b, d, f, h, j). Note the increase in EGF, NRG1 and NRG3 staining in the cytoplasm of mutant Sertoli cells compared with wild-type Sertoli cells. Similarly, the intensity of p-EGFR and ERBB4 expression was stronger in the cytoplasm of mutant spermatocytes (arrows) compared with wild-type spermatocytes (arrowheads). sc, Sertoli cells; smc, spermatocytes. Scale bar, 50 μm. (**C**) Protein levels of EGFs (EGF, NRG1 and BTC) and EGF receptors (p-EGFR and ERBB4) in isolated Sertoli cell lysate and spermatocyte lysate, respectively. β-tubulin served as the protein loading control. The data are expressed as the mean ± SEM. *p < 0.05.

EGFR (also known as ErbB1) is a member of the ErbB family of receptor tyrosine kinases, which also includes ErbB2, ErbB3 and ErbB4. Several ligands in the EGF family such as EGF, TGFα, EPGN, AREG, BTC, HB-EGF, EREG and NRG1-3, are known to specifically bind to ErbB family receptors [32]. EGF family members are secreted by Sertoli cells and that EGF receptors are present on the surface of spermatocytes [33, 34]. Previous microarray data indicate that *AR*-null testes express elevated levels of several EGF-EGFR signaling molecules, including *Egf*, *Tgf*α, *Btc* and *Erbb4* (GEO2R analysis of GEO database: GSE2259 and GSE20918) [36, 37]. To confirm the activation of EGF-EGFR signaling in SCARKO testes, we measured the mRNA levels of EGF receptor ligands and EGF receptors in isolated Sertoli cells and spermatocytes, respectively. The mRNA levels of *Egf*, *Btc* and *Nrg1* were significantly elevated in isolated Sertoli cells from SCARKO testes. In contrast, *Tgf*α, *Hbegf*, *Areg*, Ereg, *Epgn*, *Nrg2* and *Nrg3* were not differentially expressed between control and SCARKO Sertoli cells (Figure 3A a). The mRNA levels of *Egfr* and *Erbb4* were significantly up-regulated in SCARKO spermatocytes, while the expression of *Erbb2* and *Erbb3* was similar in spermatocytes from wild-type and SCARKO mice (Figure 3A b). The overexpression of EGF, BTC and NRG1 in SCARKO Sertoli cells was further confirmed by immunofluorescence in Sertoli cells isolated from wild-type and SCARKO testes (Figure 3B; a-f). Co-localization of EGFR and ERBB4 with SCP3 indicated that phosphorylated EGFR (p-EGFR) and ERBB4 were expressed at high levels and were predominantly located at the cell surface of SCARKO spermatocytes; conversely, these proteins were expressed at lower levels in control spermatocytes (Figure 3B; g-j). Moreover, the overexpression of these proteins was quantitatively confirmed by Western blot (Figure 3C). In addition, up-regulation of EGF receptors (EGFR and ERBB4) in spermatocytes is a ligand-dependent action. Because, addition of EGFR ligands (EGF, NRG1 and BTC) in ‘*in vitro* spermatocyte culture systems' could significantly up-regulate the expression of EGF receptors, including EGFR and ERBB4 (Figure S3). In summary, SCARKO testes expressed elevated levels of ligands (including EGF, BTC and NRG1) and receptors (including EGFR and ERBB4), leading to hyperactivation of EGF-EGFR signaling.

### Attenuated expression of homologous recombination factors in both SCARKO and EGF transgenic testes

**Figure 4:**
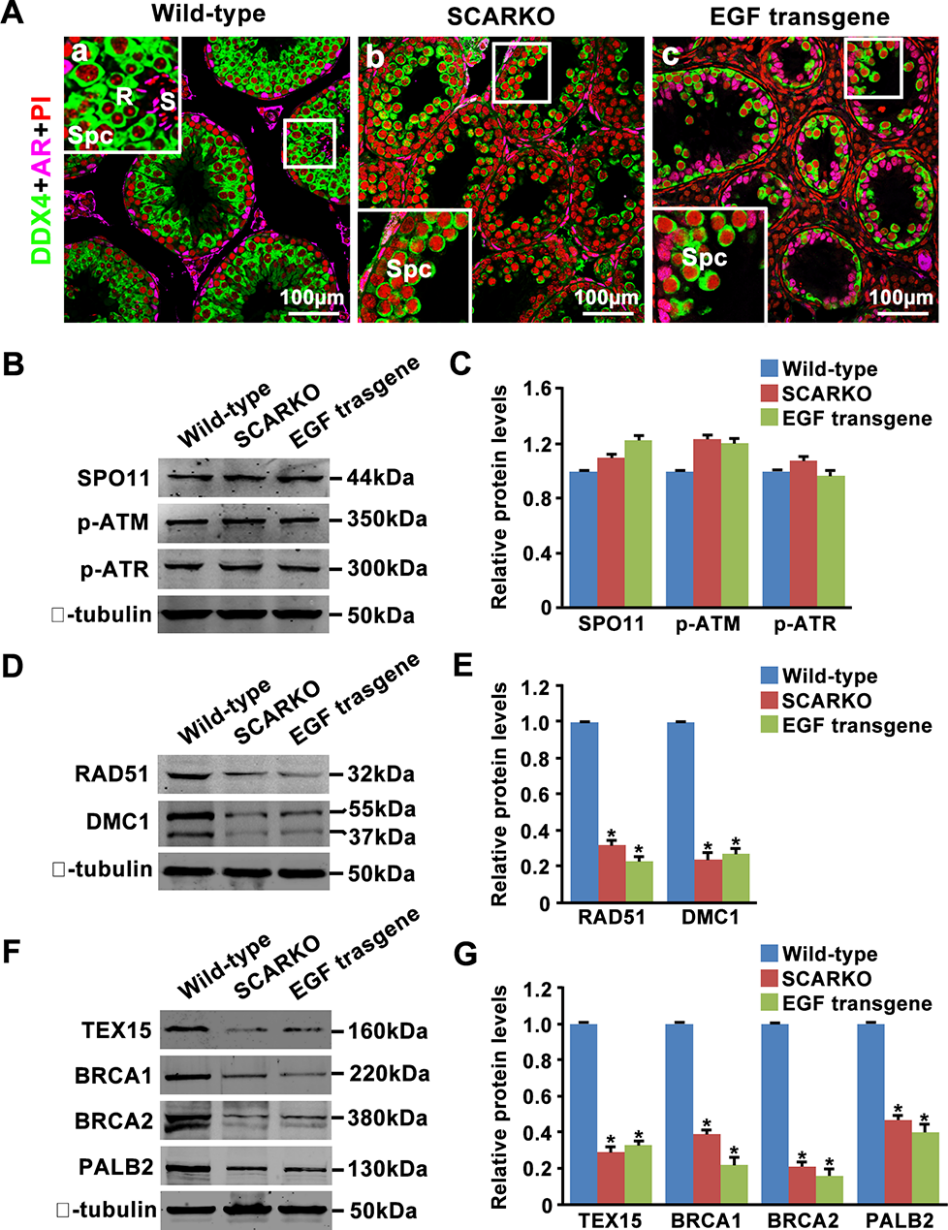
Attenuated expression of homologous recombination factors in SCARKO and EGF transgenic testis lysates (**A**) Cellular localization of DDX4 (green) and AR (pink) in sections of wild-type (a), SCARKO (b) and EGF transgenic (c) testes at postnatal day 35. The nuclei are counterstained with PI (red). R: round spermatids; S: elongated spermatids; Spc: spermatocytes. Representative images are shown from experiments that were repeated thrice using samples from different sets of testes and that yielded similar results. Scale bar, 100 μm. Western blot analysis of SPO11, p-ATM, and p-ATR (**B**, **C**); RAD51 and DMC1 (**D**, **E**); and TEX15, BRCA1, BRCA2 and PALB2 (**F**, **G**) in purified spermatocytes from postnatal day 15 wild-type, SCARKO and EGF transgenic mice. β-tubulin served as the protein loading control. The image is representative of three independent experiments. Data are expressed as the mean ± SEM. *p < 0.05.

Haploid cells (round and elongated spermatids) were produced in 35-day-old wild-type testes (Figure 4A; a). In contrast, spermatocytes underwent complete arrest in meiosis in the testes of both SCARKO mice and transgenic mice overexpressing EGF at the same age (Figure 4A; b, c). Given that SCARKO Sertoli cells expressed elevated levels of EGF (Figure 3) and that EGF transgenic mice share the meiosis arrest phenotype with SCARKO mice (Figure 4A), we focused on their common causes of marked failure during meiotic prophase I. DSBs are introduced by SPO11, while the initial detection of DSBs relies on the kinases ATM and ATR [5, 42]. We found that the protein levels of SPO11, phosphorylated ATM (Serine 1981) and phosphorylated ATR (Serine 428) in purified spermatocytes from the testes of 15-day-old mice were similar among wild-type, SCARKO and EGF transgenic mice, indicating that DSBs are formed and successfully recognized (Figure 4B, 4C). Following recognition of DSB sites, RAD51 and DMC1 are recruited to meiotic chromosomes for DSB repair [43]. Accordingly, we showed that the expression of RAD51 and DMC1 was significantly reduced in purified spermatocytes from SCARKO and EGF transgenic testes (Figure 4D, 4E). In addition, we detected the expression of the proteins that regulate RAD51 and DMC1 loading onto sites of DSBs in spermatocyte protein extracts from SCARKO and EGF transgenic testes. Notably, spermatocytes isolated from SCARKO and EGF transgenic testes expressed significantly attenuated levels of TEX15, BRCA1, BRCA2 and PALB2 compared with wild-type testes (Figure 4F, 4G). Based on these findings, we suggest that both RAD51 and DMC1 expression and TEX15-, BRCA1-, BRCA2- and PALB2-mediated RAD51 and DMC1 loading onto sites of DSBs were disrupted in SCARKO and EGF transgenic testes.

### Partial restoration of meiosis in SCARKO testes after attenuating EGF-EGFR signaling

**Figure 5:**
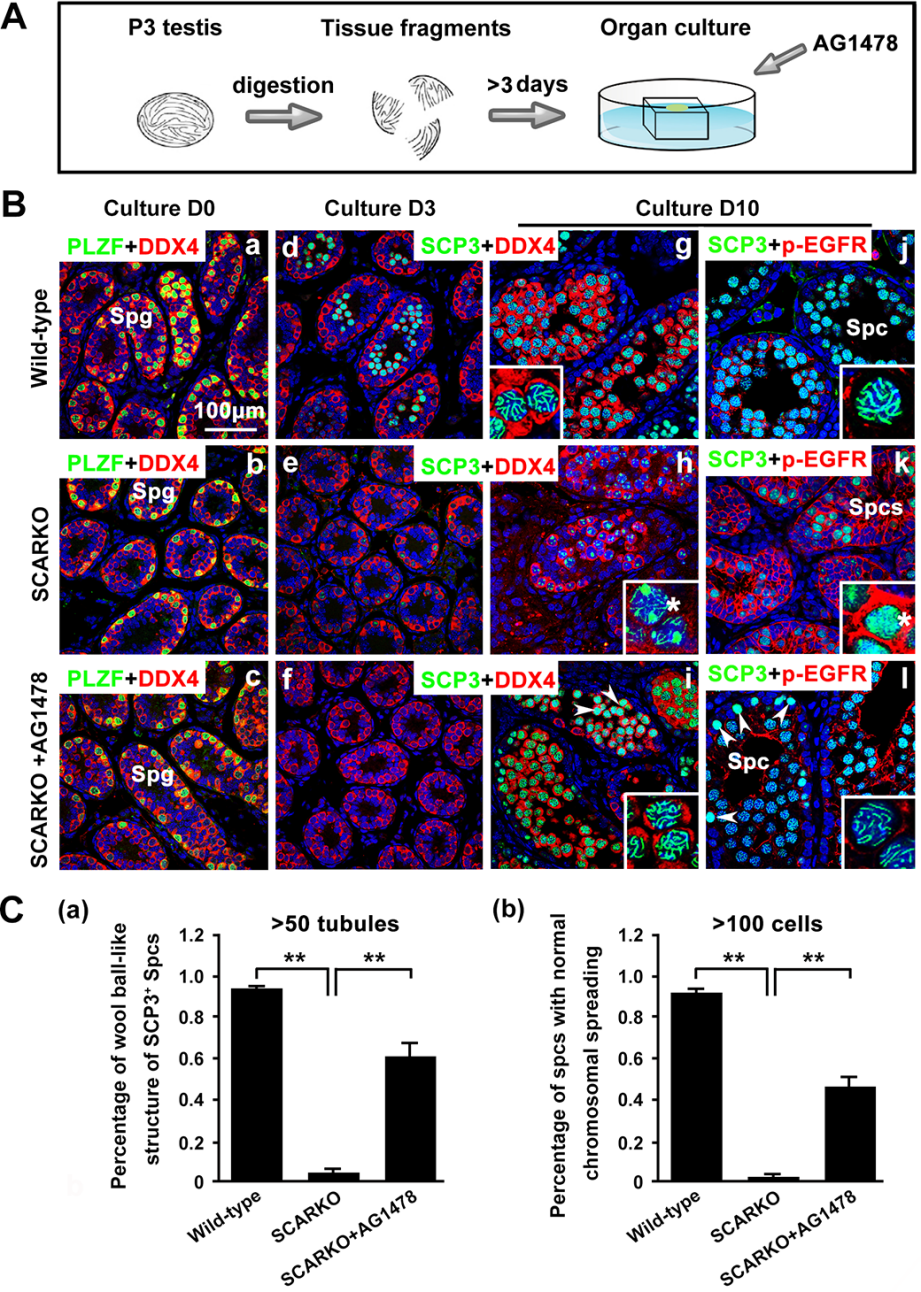
Partial restoration of defective meiosis in SCARKO spermatocytes by attenuating hyperactivated EGF-EGFR signaling (**A**) Flow chart of organ culture. Testis tissues were placed on 1.5% agarose gel stands half-soaked in medium and were cultured for 3-10 days. (**B**) Immunostaining of the spermatogonium (Smg) marker PLZF (green) and the germ cell marker DDX4 (red) in cultured testes at day 0 (a–c). Staining of the meiotic marker SCP3 (green) and DDX4 (red) in cultured testes at day 3 (d–f) and day 10 (g–i). Expression of SCP3 (green) and p-EGFR (red) in cultured testes at day 10 (j–l). Note the defective synapsis of homologous chromosomes (stars in h, k) and the hyperactivation of p-EGFR (k) in mutant spermatocytes. When the EGF-EGFR pathway in cultured SCARKO testis was repressed by the specific inhibitor AG1478, the distribution of SCP3 on mutant synapsed chromosomes was as even and clear as on wild-type chromosomes. Note that some SCARKO spermatocytes retained the meiotic defect phenotype in the rescue group (arrowheads in i, l). Scale bar, 100 μm. (**C**) Percentage of wool ball-like structure of SCP3-positive spermatocytes (> 50 tubules) (a) and the percentage of spermatocytes with normal chromsomal spreading (> 100 cells) (b) in wild-type, SCARKO and rescued groups in culture. Data are expressed as the mean ± SEM. **p < 0.01.

**Figure 6:**
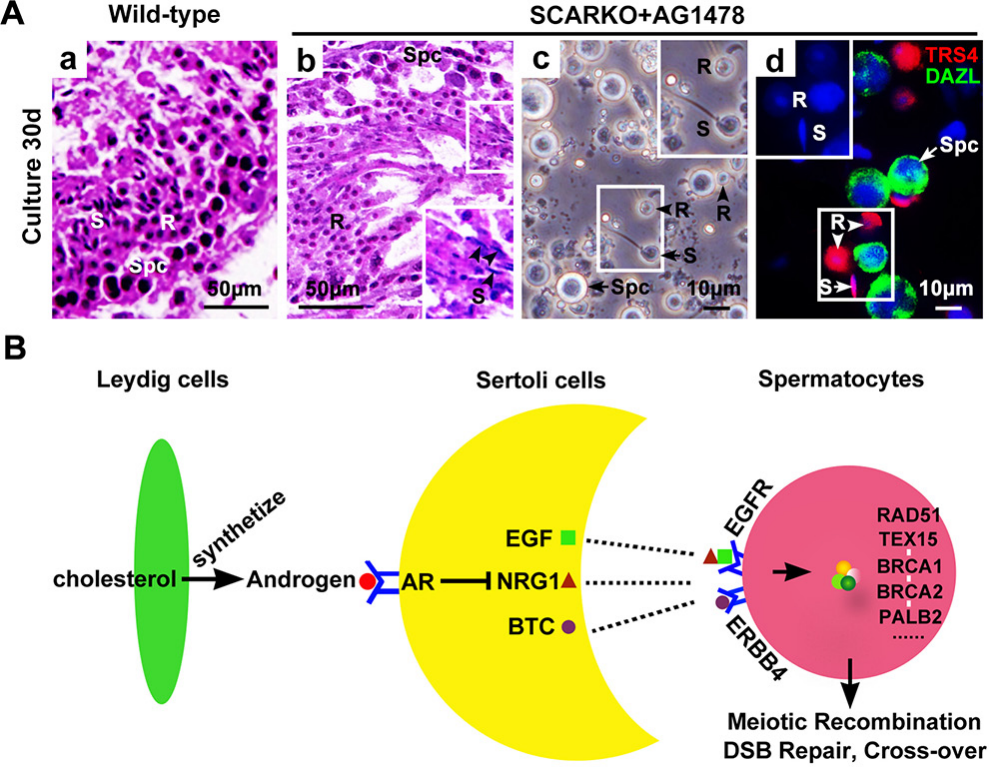
Generation of haploid sperm from AG1478-cultured SCARKO testis tissues *in vitro* (**A**) Tissues sections from neonatal wild-type (a) and SCARKO mice (b) showed representative seminiferous tubules with spermatogenesis after thirty days in culture. The black arrowheads in b indicate elongated spermatids. Spermatocytes as well as round and elongated spermatids were present in cultured samples of AG1478-treated SCARKO testis after mechanical dissociation of the cells (c). Immunostaining with anti-TRS4 (red) and anti-DAZL (green) antibodies and counterstaining with DAPI (blue) (d). S: elongated spermatids; R: round spermatids; Spc: spermatocytes; B: blastocyst; O: oocyte. Scale bars, 50 μm (a, b) and 10 μm (c, d). (**B**) Possible mechanism of meiotic initiation by AR in Sertoli cells through activation of intercellular EGF-EGFR signaling. Leydig cells in the interstitial region synthesize the androgens from cholesterol through a series of steroid enzymes. Androgens function in Sertoli cells via binding and activation to AR to (directly or indirectly) regulate the expression of EGFs, including *Egf*, *Btc* and *Nrg1*. These EGF family ligands directly act on spermatocytes via their corresponding receptors, including EGFR and ERBB4, to stimulate the expression and accumulation of homologous recombination factors, including RAD51, TEX15, BRCA1/2 and PALB2. Thus, androgen from Leydig cells and AR in Sertoli cells can ultimately induce chromosomal synapsis and meiotic recombination repair in spermatocytes.

To determine whether elevated EGF-EGFR signaling caused the observed meiosis defects in SCARKO males, the EGFR phosphorylation-inhibitor AG1478 was added to the postnatal day (P) 3 SCARKO testis tissue culture system. Spermatocyte meiosis progression was detected in SCARKO testes after 3 days of treatment (Figure 5A). We observed that the membrane expression (Figure 5B; j-l) and protein level (Figure S4A) of p-EGFR could be repressed to approximately normal (compared with wild-type) by the 200 μM AG1478 in the cultured SCARKO testis tissues. The cultured wild-type testis tissues were stained for SCP3, showing a representative chromosomal expression pattern (wool ball-like structure) in spermatocytes (Figure 5B; g, j). The distribution of SCP3 on synapsed chromosomes was uneven in SCARKO spermatocytes (Figure 5B; h, k). Conversely, wool ball-like structure of SCP3-positive spermatocytes was observed in inhibitor-treated SCARKO testis tissues (Figure 5B; i, l). The percentage of wool ball-like structure of SCP3-positive spermatocytes was significantly up-regulated in inhibitor-treated SCARKO testes, compared with SCARKO testes (> 50 tubules) (Figure 5C; a). Then, the cultured testis tissues were dissociated and stained with meiosis-associated markers, including SCP1, SCP3, CREST, γ-H2AX and RAD51 (Figure S4B). The meiotic defects in SCARKO testis tissues were consistent with the above results (Figures 1 and 2), with aberrant chromosomal synapsis and unrepaired DSBs after 10 days in culture (Figure S4B; a, c, e, g). In contrast, inhibitor-treated SCARKO spermatocytes showed (i) intact accumulation and co-localization of SCP1 and SCP3 around the whole synaptonemal complex (Figure S4B; b and C; a); (ii) no univalent chromosomes (CREST foci=20) (Figure S4B; d and C; b); (iii) undetectable γ-H2AX staining on autosomes but detectable staining restricted to the largely asynapsed XY body (Figure S4B; f and C; c); and (iv) more abundant RAD51 foci on the chromosomes (Figure S4B; h and C; d). The percentage of spermatocytes with normal chromsomal spreading was significantly up-regulated in inhibitor-treated SCARKO testes, compared with SCARKO testes (> 100 cells) (Figure 5C; b). After a 30-day culture, we found typical round and elongated spermatids in cultured SCARKO testis samples treated with 200 μM AG1478 (Figure 6A; b). These findings were further supported by the observation of TRS4-positive round and elongated spermatids after mechanical dissociation of the inhibitor-treated SCARKO tissues into a cell suspension (Figure 6A; c, d). Collectively, these results demonstrated that incubation with 200 μM EGFR phosphorylation-inhibitor AG1478 partially restores meiotic defects of some SCARKO spermatocytes.

## DISCUSSION

Despite progress in understanding the importance of AR expression in Sertoli cells on spermatocyte meiosis, the details and underlying mechanisms are currently unclear. In this study, we ascertained which steps of meiotic prophase I were affected by the absence of AR in Sertoli cells. We utilized co-immunostaining of meiotic surface spreads to show that chromosomal synapsis (Figure 1) and DSB repair (Figure 2) were impaired in SCARKO spermatocytes.

During meiotic prophase I, DSBs are generated by the type II-like topoisomerase SPO11 [42]. In response to DSBs, ATM/ATR “raise the alarm” to indicate DNA damage, phosphorylating many downstream effectors and opening the chromatin structure to allow access to the repair machinery [44]. Then, TEX15, BRCA1, BRCA2 and PALB2 mediate loading of the RAD51 and DMC1 recombinases onto sites of DSBs [7–10]. We showed here by Western blotting analysis that normal levels of p-ATM and p-ATR but low levels of TEX15, BRCA1, BRCA2 and PALB2 were present in SCARKO spermatocytes, indicating that the generation of and response to DSBs occurs normally but that these DSBs are not repaired efficiently. In addition to RAD51 loading, the protein levels of RAD51 and DMC1 were also attenuated in SCARKO testes. Thus, homologous recombination-mediated repair of DSBs is impaired in SCARKO testes due to deficiencies in both the expression and recruitment of homologous recombination factors such as RAD51 and DMC1, leading to asynapsis. The phenotype of the SCARKO testes is reminiscent of other mouse mutants in which defective homologous recombination leads to aberrant chromosomal synapsis and impaired DSBs [45–47]. Protein expression analyses of these factors may be helpful to gain further insight into the regulatory mechanisms in SCARKO spermatocytes.

Sialoadenectomy reduces the amount of circulating EGF to an undetectable level and thereafter results in a dramatic decrease in epididymal sperm storage [48, 49]. On the other hand, overexpression of EGF induces infertility in transgenic mice [35]. Thus, we believe that proper EGF expression is required for the normal completion of spermatogenesis. In this study, we observed that EGF-EGFR signaling was hyperactivated in SCARKO testes. Moreover, the meiotic arrest phenotype observed in SCARKO meiocytes is very similar to that in meiocytes that overexpress EGF in the transgenic mouse [35]. Similar to SCARKO testes, which expressed elevated EGF, the expression of homologous recombination factors, including RAD51, DMC1, TEX15, BRCA1/2 and PALB2, was attenuated in EGF transgenic testes. Accordingly, we suggest that AR negatively regulates EGF, which when over-expressed, suppresses the expression of these homologous recombination factors. Our finding that AR negatively regulates *Egf* expression in Sertoli cells could suggest a possible link between AR signaling and the EGF-EGFR pathway. However, the underlying mechanism by which AR regulates EGF (directly or indirectly) requires further investigation. In addition, the overlapping gene profiles in SCARKO and EGF-overexpressing meiocytes must be examined in future studies. An understanding of the molecular mechanisms by which androgens drive spermatogenesis has been thwarted by the fact that different studies identified many different candidate AR target genes [36, 37, 50, 51]. Differences of animal model, ages and detection methods among these studies may account for their different gene profile.

Based on all our findings, we suggest a model in which AR in Sertoli cells transduces the androgenic stimulus to spermatocytes partially via intercellular EGF-EGFR signaling (Figure 6B). Leydig cells in the interstitial region synthesize the androgens from cholesterol through a series of steroid enzymes. Androgens function in Sertoli cells via binding and activation to AR to (directly or indirectly) regulate the expression of EGFs, including *Egf*, *Btc* and *Nrg1*. These EGF family ligands directly act on spermatocytes via their corresponding receptors, including EGFR and ERBB4, to stimulate the expression and accumulation of homologous recombination factors, including RAD51, TEX15, BRCA1/2 and PALB2. Thus, androgen from Leydig cells and AR in Sertoli cells can ultimately induce chromosomal synapsis and meiotic recombination repair in spermatocytes.

We attempted to rescue the meiotic arrest phenotype of the SCARKO testes by moderately attenuating the hyperactivated EGF-EGFR signaling *in vitro*. We utilized an ‘*in vitro* testis tissue culture system’, established by Sato et al. [52], and supplemented the medium with 200 μM tyrphostin AG1478. After thirty days of treatment, spermatogenesis progressed beyond meiosis in SCARKO testis tissues, thus generating haploid sperm. We term this ‘partial’ rescue as opposed to complete rescue, as approximately half of the meiocytes (100 cells counted from 3 males) showed continued failure of chromosomal synapsis and DSB repair after inhibitor treatment. Other factors or signaling events potentially act as downstream effectors of AR signaling and must be activated or suppressed. Although the efficiency of haploid sperm generation was relatively low, and other disordered factors or signaling events require modification, our rescue experiments may provide a new lead for the treatment of infertile patients carrying *AR* mutation.

In summary, our results show that meiotic prophase progression, especially chromosomal synapsis and DSB repair, must be strictly controlled by proper Sertoli-secreted EGF signals that are negatively regulated by AR. Importantly, treatment with 200 μM AG1478, which inhibits EGFR phosphorylation, partially restored meiosis and generated haploid sperm.

## MATERIALS AND METHODS

### Mouse strains

All experimental protocols and animal handling procedures were conducted in accordance with the guidelines and procedures approved by the Institutional Animal Care and Use Committee (IACUC) of the Institute of Zoology (IOZ), University of Chinese Academy of Sciences (UCAS). All the mice were maintained in a C57BL/6;129/SvEv mixed background. We obtained both floxed AR mice (stock no: 018450) and *Amh*-Cre transgenic mice (stock no: 007915) from The Jackson Laboratory (donating investigator: Robert E Braun). By mating floxed AR mice [53] with a transgenic line possessing *Amh* promoter-driven expression of Cre recombinase [54], we obtained male S-AR^−/y^ (hereafter referred to as SCARKO) mice with the AR gene deleted in only Sertoli cells. EGF transgenic mice were generated using the β-actin promoter to drive EGF expression [35]. DNA isolated from tail biopsies was used for genotyping. Genotyping was performed by PCR as described previously [22, 35].

### Immunofluorescence analysis

Mice were euthanized via cervical dislocation, and the testes were immediately fixed in 4% formaldehyde (PFA) in PBS for immunostaining, as previously described [55]. In brief, tissue sections were deparaffinized and rehydrated, and antigen retrieval was performed in 10 mM sodium citrate buffer. For immunofluorescence, the sections were blocked with blocking buffer (donkey serum, 0.3% Triton X-100 in PBS) and incubated with primary antibodies overnight at 4°C. Sections were washed and incubated with FITC- or TRITC-conjugated secondary antibodies (Jackson ImmunoResearch, CA, USA) for 1 hour and counterstained with DAPI or PI (Sigma, MO, USA) to identify the nuclei. Images were visualized using a microscope (Axioskop 40; Carl Zeiss), captured with a digital camera (Zeiss AxioCam MRc5), and processed with Photoshop (Adobe). The primary antibodies used for immunostaining are listed in Table S1.

### Meiotic chromosome spreads

Spreads were prepared by the dry-down technique as previously described [56]. Briefly, testis tissues were dissected, and tubules were dissociated with a pipet in PBS. Cells were ruptured by adding an equal amount of hypotonic buffer (30 mM Tris-HCl pH 8.2, 50 mM sucrose, 17 mM sodium citrate, 5 mM EDTA, 0.5 mM DTT, and protease inhibitors) and then mixing the solution 1:2 with 100 mM sucrose just before spreading on slides that were pre-incubated with 1% PFA. The slides were then dried for 1 hour. Immunostaining of spermatocyte spreads was performed similar to the immunofluorescence analysis.

### Isolation of sertoli cells and spermatocytes

We modified a previously described method to isolate Sertoli cells from the testes of 3-week-old mice [57, 58]. Briefly, the seminiferous tubules were pooled and incubated with 1 mg/ml collagenase IV (Sigma), 1 mg/ml hyaluronidase (Sigma) and 0.5 mg/ml DNase I (Sigma) in DMEM/F12 medium (HyClone) for 5 minutes at 37°C in a shaker. These dispersed cells were cultured and then treated with a hypotonic solution (20 mM Tris, pH 7.4) for 1 minute to remove the remaining germ cells. The purity of Sertoli cells was confirmed by quantitative RT-PCR of *Wt1* (Sertoli cell marker), *Mvh* (Germ cell marker), *Cyp11a1* (Leydig cell marker) and *Myh11* (Peritubular myoid cell marker) (Figure S2). Spermatocytes were isolated by enzymatic dissociation of testes from 3-week-old mice, and the cells were separated by gravity sedimentation in a 2–4% BSA gradient in a STA-PUT apparatus (ProScience Inc) according to the method described by La Salle et al. [59].

### Western blot analysis

Western blot analysis was performed as described previously [58]. Proteins from isolated Sertoli cells and purified spermatocytes were electrophoresed under reducing conditions in 12% SDS-PAGE gels and transferred to nitrocellulose membranes. The blots were blocked in 5% BSA, incubated overnight at 4°C with the primary antibody, and incubated with the IRDye 680 or IRDye 800 (LI-COR) secondary antibody for 1 hour at room temperature. The specific signals and the corresponding band intensities were evaluated using an Odyssey Infrared Imaging system and software (LI-COR Bioscience). The primary antibodies used for the Western blot analysis are listed in Table S1.

### Quantitative RT-PCR

RNA was extracted using Trizol (Invitrogen, TX, USA) according to the manufacturer's protocol. RNA samples were subjected to reverse transcription using a PrimeScript RT Reagent Kit (Takara, Dalian, China). The reactions were run in triplicate in three independent experiments. The CT values for the samples were normalized to the corresponding *Gapdh* CT values, and relative expression levels were calculated using the ΔΔCT method. The primer sequences are provided in Table S2.

### Organ culture

The testes from neonatal wild-type and SCARKO males were cut into several pieces, 1 to 3 mm in diameter. Agarose gel stands (1.5% (w/v)) were prepared 1 day before initiating the culture and were incubated with culture medium for more than 24 hours. Testis explants were placed at the medium-air interface on each agarose gel stand. The culture media was reported in a previous study [52] and was supplemented with 200 μM Tyrphostin AG1478 (S2728; Selleck) or vehicle. Medium changes were performed every two days. The incubator was supplied with 5% CO_2_ and 95% air and was maintained at 33°C. For *in vitro* spermatocyte culture systems, testes from postnatal day 3 pups were digested into single cells and were cultured in DMEM supplemented with 10% FBS, 1% non-essential amino acid, 1% L-glutamine for 15 days. Recombinant murine EGF, NRG1 or BTC (100 μg each, all purchased from Perprotech, USA) were added to the culture media or not.

### Tissue collection and histological analysis

Testis explants were collected after forty days of *in vitro* organ culture, fixed in 4% PFA for up to 24 hours, stored in 70% ethanol, and embedded in paraffin. Sections (5-μm thick) were cut and mounted on glass slides. After deparaffinization, sections were stained with hematoxylin-eosin (H & E) for histological analysis.

### DNA-dependent protein kinase (DNA-PK) assays

DNA-PK pulldown kinase assays were conducted as previously described [39]. Extract was incubated with 20 μl of preswollen double-stranded DNA (dsDNA)-cellulose (Affymetrix, 14394, Beijing, China) in a total volume of 50 μl of binding buffer (25 mM Hepes/KOH at pH 7.9, 50 mM KCl, 10 mM MgCl_2_, 20% glycerol, 0.1% Nonidet P-40, 1 mM dithiothreitol) for 4 hr on ice. The unbound fraction was removed by centrifugation, and the dsDNA-cellulose was washed three times and resuspended in 50 μl of binding buffer. 0.5 μl of [γ-^32^P]ATP was added, and kinase assays were conducted in the presence or absence of 4 nmol of peptide (0.2 mM). Reactions were then stopped and analyzed by liquid scintillation counting. The sequences of wild-type and mutant p53 peptides are EPPLSQEAFADLLKK and EPPLSEQAFADLLKK, respectively.

### Statistical analysis

Experiments were repeated at least three times. The data were evaluated for significant differences using Student's *t*-test. The results are presented as the mean ± SEM. Statistical significance was considered at *p < 0.05 and **p < 0.01.

## SUPPLEMENTARY MATERIALS FIGURES AND TABLES


