# Supplementary material for: Androgen receptor in Sertoli cells regulates DNA double-strand break repair and chromosomal synapsis of spermatocytes partially through intercellular EGF-EGFR signaling
Source: Oncotarget. 2016 Mar 4;7(14):18722–35. doi: 10.18632/oncotarget.7916 (PMC4951324; doi:10.18632/oncotarget.7916)
Supplement: Supplementary file 1 [file oncotarget-07-18722-s001.pdf]

Androgen receptor in sertoli cells regulates DNA double-strand break repair and chromosomal synapsis of spermatocytes partially through intercellular EGF-EGFR signaling

Supplementary Materials

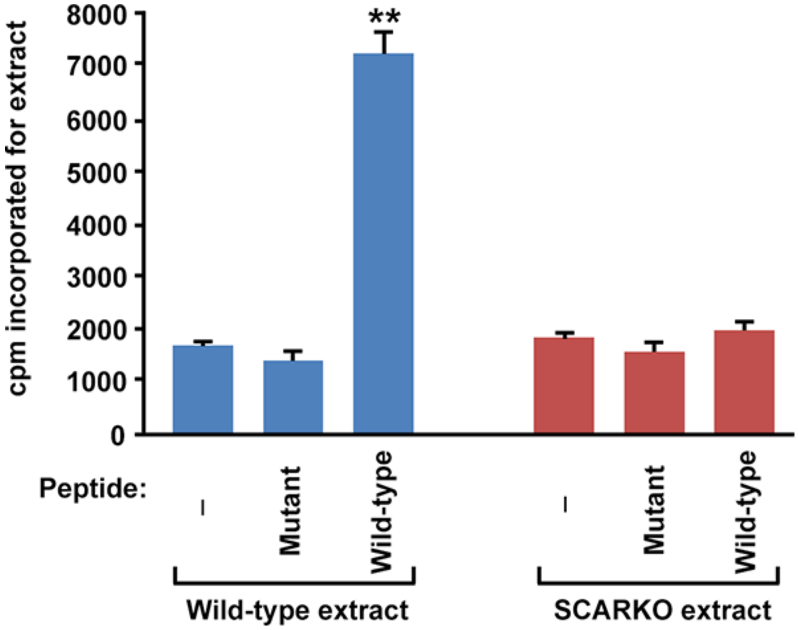

Supplementary Figure S1: The DNA-PK pulldown peptide assay detects DNA-PK in spermatocyte extracts. Whole cell extract (150 µg) from wild-type or SCARKO spermatocytes was used in a DNA-PK pulldown peptide phosphorylation assay with wild-type or mutant p53 peptide or in the absence of peptide (−). Data are expressed as the mean ± SEM. ***p* < 0.01.

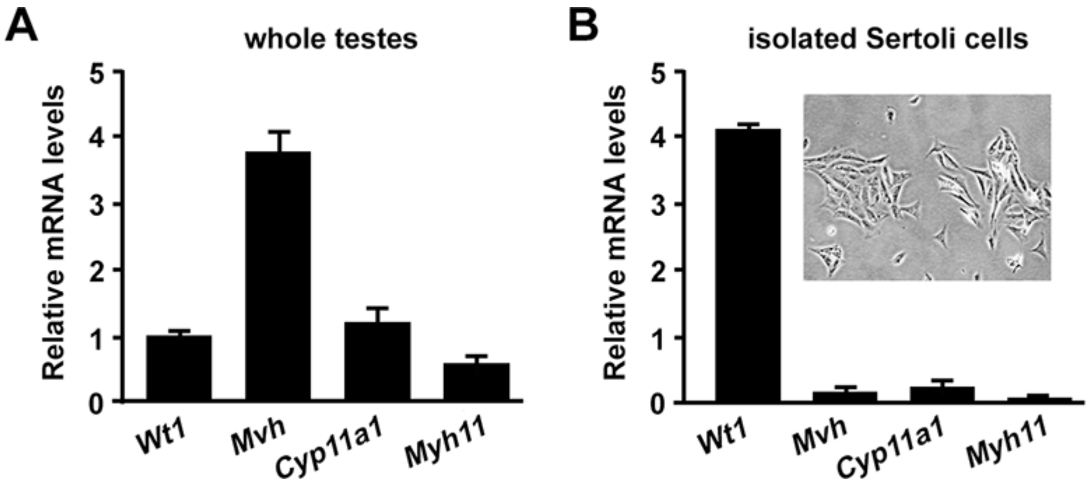

Supplementary Figure S2: High-purity of sertoli cells. The purity of Sertoli cells was confirmed by qRT-PCR of *Wt1* (Sertoli cell marker), *Mvh* (Germ cell marker), *Cyp11a1* (Leydig cell marker) and *Myh11* (Peritubular myoid cell marker) in both whole testes (**A**) and isolated Sertoli cells (**B**).

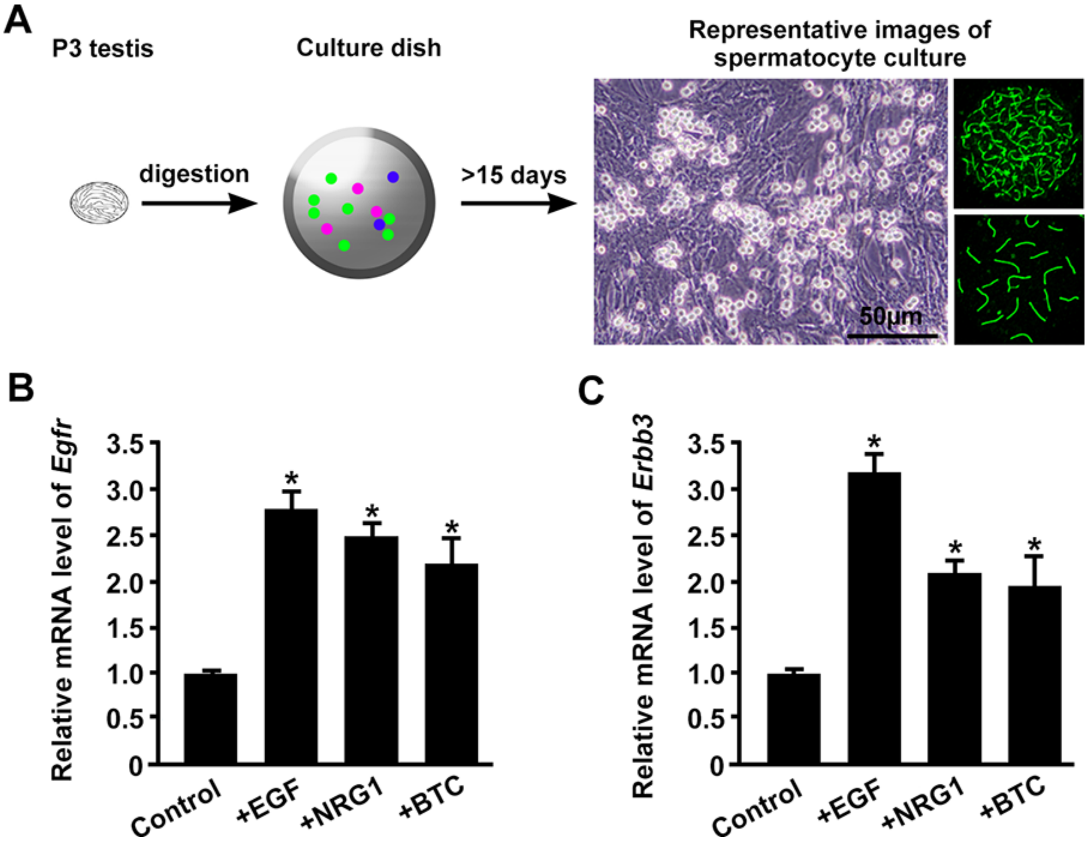

Supplementary Figure S3: Up-regulation of EGF receptors in spermatocytes is a ligand-dependent action. (**A**) Model for *in vitro* spermatocyte culture systems and representative images of spermatocyte culture. (**B**, **C**) The mRNA levles of EGF receptors (EGFR and ERBB4) after addition of EGFR ligands (EGF, NRG1 and BTC) in '*in vitro* spermatocyte culture systems'. Data are expressed as the mean ± SEM. $*p < 0.05$.

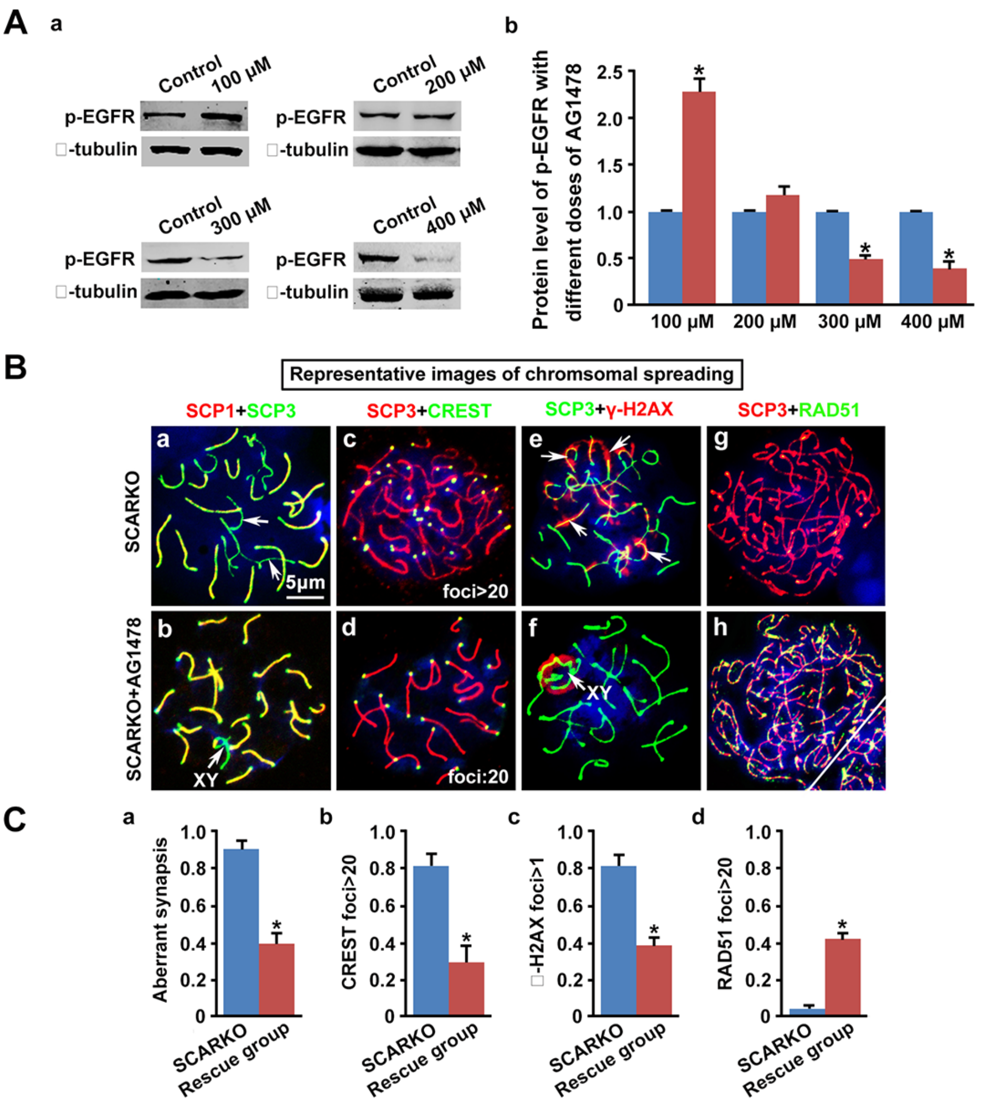

Supplementary Figure S4: EGFR phosphorylation-inhibitor AG1478 (200 µM) partially restored meiosis. (**A**) The protein level of p-EGFR in cultured SCARKO testis tissues by different doses (100 µM~400 µM) AG1478 treatment for 3 days. (**B**) Representative images of chromsomal spreading of spermatocytes from cultured SCARKO and 200 µM inhibitor-treated SCARKO testis tissues, stained with meiosis-associated markers, including SCP1, SCP3, CREST, γ-H2AX and RAD51. Arrows in a and e indicate unpaired chromosomes and γ-H2AX foci on asynapsed autosomal homologs, respectively. Scale bar, 5 µm. (**C**) Percentage of spermatocytes with aberrant synapsis (a), CREST foci > 20 (b), γ-H2AX >1 (c) and RAD51 foci > 20 (d) in SCARKO and rescue group (200 µM inhibitor-treated SCARKO). Data are expressed as the mean ± SEM. *$p < 0.05$.

Supplementary Table 1: Primary and secondary antibodies used in this study

Mouse anti-SCP3	1/400	Abeam	ab97672	IF
Rabbit anti-SCP1	1/400	Abeam	ab15090	IF
CREST antiserum	1/400	Antibodies Inc	15-234-0001	IF
Rabbit anti-yH2AX	1/400	Abeam	ab11174	IF
Rabbit anti-RAD51	1/500	Millipore	ABE257	IF, WB
Rabbit anti-MLH1	1/400	Abeam	ab92312	IF
Rabbit anti-EGF	1/400	Abeam	ab9695	IF, WB
Rabbit anti-NRG1	1/400	Abeam	ab27303	IF, WB
Rabbit anti-NRG3	1/400	Abeam	ab109256	IF
Goat anti-p-EGFR	1/400	Epitomics	1727-1	IF, WB
Mouse anti-ERBB4	1/500	Millipore	05-1133	IF, WB
Rabbit anti-DDX4	1/400	Abeam	ab13840	IF
Mouse anti-AR	1/400	Abeam	ab9474	IF
Mouse anti-PLZF	1/200	Santa Cruz	se-28319	IF
Rabbit anti-SP011	1/1000	Abeam	ab81695	WB
Mouse anti-p-ATM	1/500	Cell Signaling	#4526	WB
Rabbit anti-p-ATR	1/500	Cell Signaling	#2853	WB
Goat anti-DMC1	1/500	Santa Cruz	sc-8973	WB
Rabbit anti-TEX15	1/1000	Proteintech	24585-1-AP	WB
Rabbit anti-BRCA1	1/500	Santa Cruz	se-642	WB
Rabbit anti-BRCA2	1/500	Santa Cruz	se-28235	WB
Goat anti-PALB2	1/500	Santa Cruz	sc-160647	WB
Rabbit anti-DAZL	1/500	AbD Serotec	MCA2336	IF
Rabbit anti-TRS4	1/200	Our lab	-	IF
Rabbit anti-p-tubulin	1/1000	Abeam	ab6046	WB

Supplementary Table 2: Primers used in this study

Egf	Forward:	AGCATCTCTCGGATTGACCCA	Reverse:	CCT GTCCCGTTAAGGAAAACT CT
Hbegf	Forward:	CGGGGAGTGCAGATACCTG	Reverse:	TTCTCCACTGGTAGAGTCAGC
Tgfa	Forward:	CACTCTGGGTACGTGGGTG	Reverse:	CACAGGTGATAATGAGGACAGC
Areg	Forward:	GGTCTTAGGCTCAGGCCATTA	Reverse:	CGCTTATGGTGGAAACCTCTC
Ereg	Forward:	CTGCCT CTTGGGTCTT G ACG	Reverse:	GCGGTACAGTTATCCTCGGATTC
Epgn	Forward:	CAGAAGCCAAGTT CACTA	Reverse:	CTTTGCTATTAG GT CC AT
Nrg1	Forward:	ATGGAGATTTATCCCCCAGACA	Reverse:	GTT GAGGCACCCT CT GAG AC
Nrg2	Forward:	GGATGGCAAGGAACTCAACC	Reverse:	T CGGCCT CACAG ACGTACT
Nrg3	Forward:	TTACGCT GTAGCGACTGCAT C	Reverse:	GCCTACCACGATCCATTTAAGC
Nrg4	Forward:	CACGCTGCGAAGAGGTTTTTC	Reverse:	CGCGATGGTAAGAGTGAGGA
Egfr	Forward:	GCCATCTGGGCCAAAGATACC	Reverse:	GTCTTCGCATGAATAGGCCAAT
Erbb2	Forward:	GAGACAGAGCTAAGGAAGCT GA	Reverse:	ACGGGGATTTTCACGTTCTCC
Erbb3	Forward:	AAGT G ACAGGCTAT GTACTGGT	Reverse:	GCTGG AGTTGGTATT GTAGTT CA
Erbb4	Forward:	GTGCTATGGACCCTACGTTAGT	Reverse:	T C ATT G AAGTT CAT G C AGG C AA
Wt1	Forward:	GAGAGCCAGCCTACCATCC	Reverse:	GGGTCCTCGTGTTTGAAGGAA
Mvh	Forward:	ATGTCT G CC AC AACTT CT GAG	Reverse:	CT G ATTTCGGTTT CATCCATCCT
Cyp11a1	Forward:	AGGTCCTTCAATGAGATCCCTT	Reverse:	TCCCTGTAAATGGGGCCATAC
Myh11	Forward:	AAGCTGCGGCTAGAGGTCA	Reverse:	CCCTCCCTTT G ATGGCT GAG
Gapdh	Forward:	TGGATTTGGACGCATTGGTC	Reverse:	TTTGCACTGGTACGTGTTGAT
